# The electronic origins of the “rare earth” texture effect in magnesium alloys

**DOI:** 10.1038/s41598-021-93703-w

**Published:** 2021-07-08

**Authors:** Reza Mahjoub, Nikki Stanford

**Affiliations:** Future Industries Institute, University of South Australia, Mawson Lakes, SA 5095 Australia

**Keywords:** Condensed-matter physics, Structural materials, Theory and computation

## Abstract

Although magnesium alloys are lightweight, recyclable and relatively cheap, they suffer from poor ductility. This can be improved by the addition of rare earth (RE) elements, and this is now a well-established criterion for wrought alloy design. It is notable that this behavior is largely restricted to the lanthanides, but no hypothesis is yet available to explain why other elements do not have the same effect. To answer this question, ab initio simulations of crystallographically complex boundaries have been undertaken to examine the electronic origin of the RE effect. While the electronic structure provided strong bonding between the RE elements and their Mg surroundings, local disruption in atomic arrangement at the grain boundaries was found to modify this effect. This work shows quantifiable changes in electronic structure of solutes resulting from grain boundary crystallography, and is suggested to be a contributing factor to the RE texture effect.

## Introduction

### Experimental background

The ductility^1^ and formability^2–4^ of magnesium alloys is known to be significantly improved by grain size refinement^5,6^ and a change in the crystallographic preferred orientation^1^. The preferred orientation, commonly referred to as the “texture”, is a statistical measure of the alignment of the grains within a material. The ductility of magnesium alloys is more sensitive to texture variations than other metals, with a randomization of the texture being one of the key goals of alloy design. This is achieved by the addition of one of the rare earth (RE) elements, as these elements are known to be potent texture modifiers during recrystallisation. This is a general behaviour observed in the Lanthanide series of elements on the periodic table, along with Y and Ca. This is commonly referred to in the literature as the “rare earth effect”.

It has been shown experimentally that RE elements segregate to dislocations^7^ and grain boundaries^8–17^, and it is the premise of the present work that the segregation of these solutes to the boundary are responsible for recrystallisation texture development^14^. It is our hypothesis that solutes behave differently at different types of grain boundaries. Consequently, the boundary properties such as energy and mobility will be changed when solute is present, resulting in different grain orientations being preferred during recrystallisation. Why such small concentrations of these elements, in the ppm range^18^ can have such a potent effect still remains unclear.

**Figure 1:**
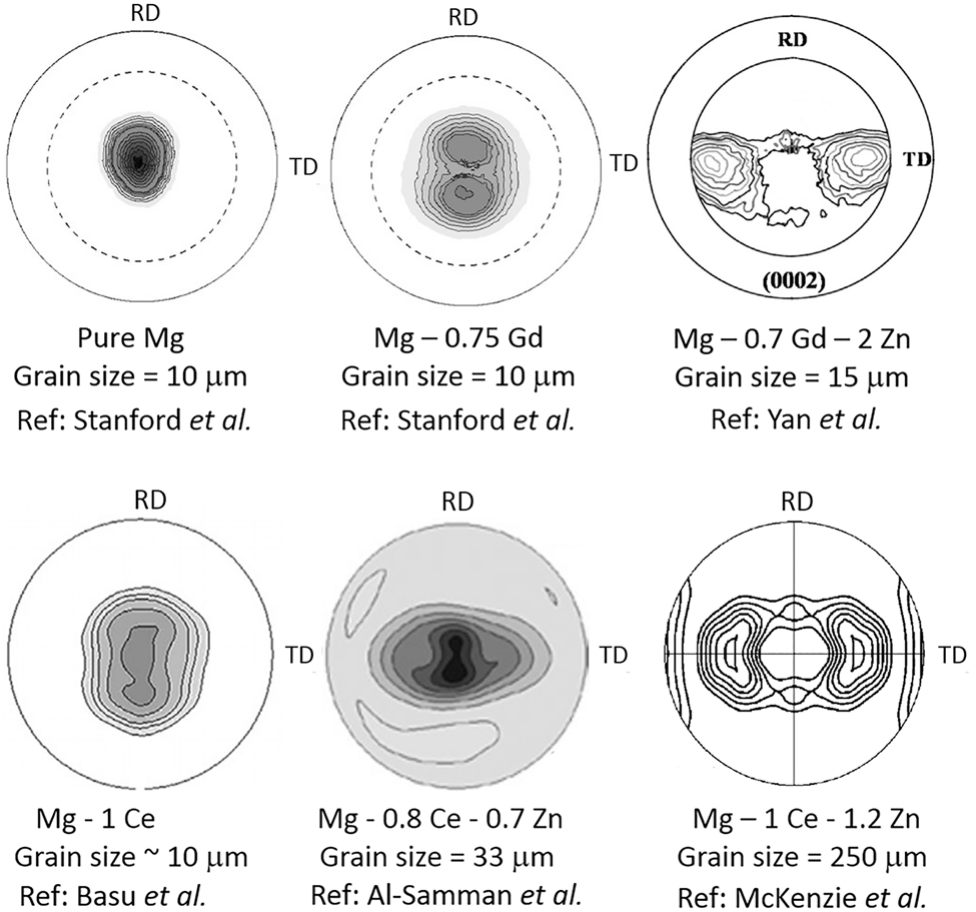
Literature review of ternary alloy behaviour^19–23^. All pole figures are of the (0001) plane and are shown with respect to the rolling direction (RD) and transverse direction (TD).

It has also been found that Zn has a synergistic effect with the RE elements, modifying the texture even further. This can be seen in Fig. 1 where the addition of Gd weakens the texture^19^ (Fig. 1a,b), but only when both Gd and Zn are present together orientation of maximum intensity moves from the centre of the stereographic projection (Fig. 1a,b) towards the transverse direction by a significant amount (Fig. 1c)^20^. The same is found for Mg-Ce and Zn (Fig. 1d–f)^21–23^. Although there has not been much speculation in the literature about why this synergistic effect may occur, co-segregation of Zn and RE elements have been observed at symmetrical boundaries^8^, and also at general grain boundaries.

There has been much speculation about the origin of this texture modification, and it has been suggested that the large atomic size of these elements causes the RE effect^18^. Although atomic size explains the energetic reasons for these elements to segregate to the grain boundary, it doesn’t explain how their presence changes the preferential growth of certain boundary types. Particularly perplexing is the restriction of this phenomena to the RE group within the periodic table, there is clearly something about the electronic structure of these elements that gives them different properties compared to others when alloyed with magnesium. Despite this clear indication that there are important chemical factors at play, the quantum mechanical behaviours of these elements in magnesium have not before been interrogated in depth.

### Overview of the present work

**Figure 2:**
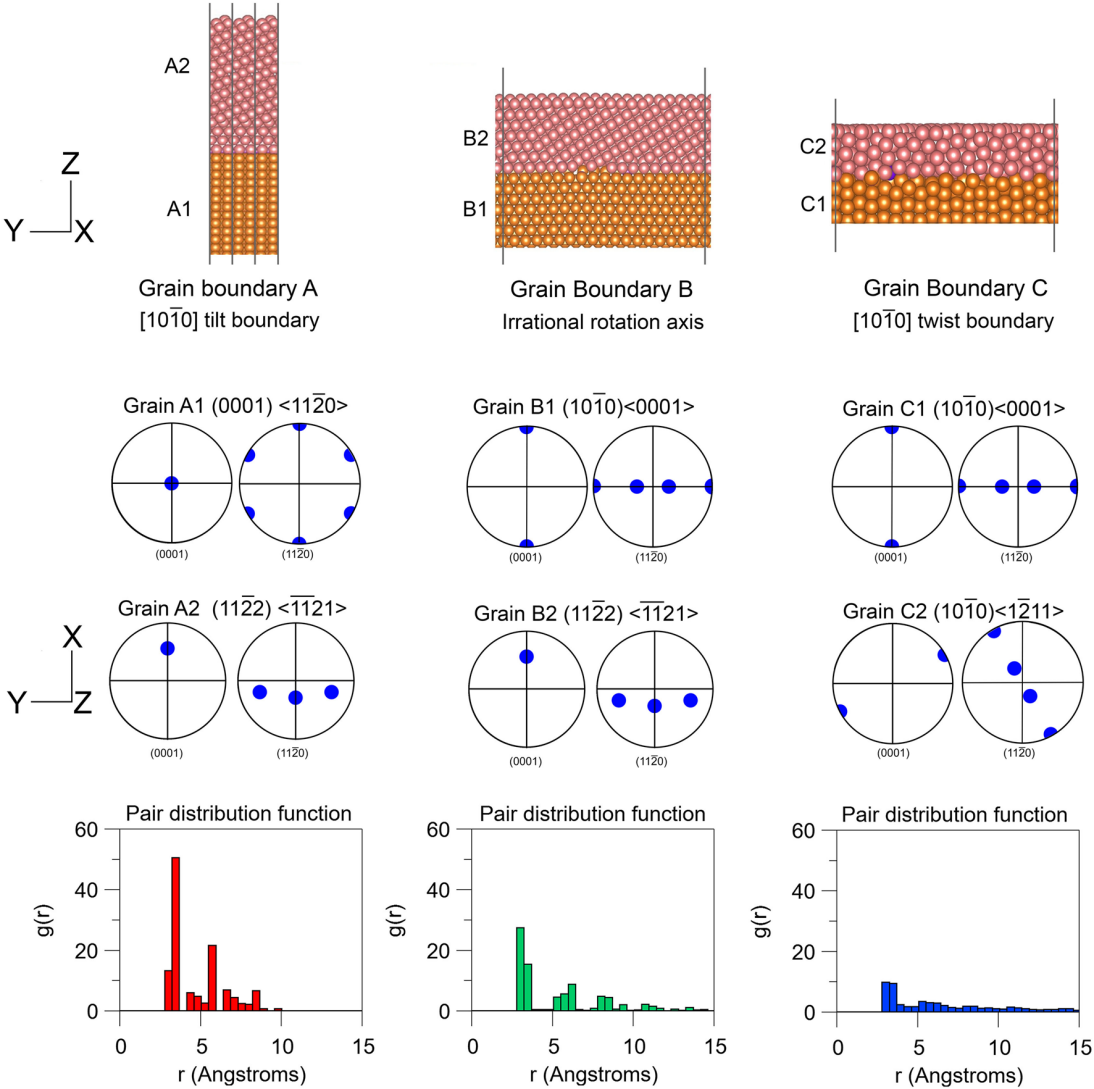
The simulation cells for the three grain boundaries examined here. The visualization was carried out using VESTA program^25^.

To this end we now move to atomic scale grain boundary simulations to help us understand these experimental findings in more detail. In the present case we utilize first principles calculations, as this provides us with the ability to add different solute species into the simulation and provides a full electron description of the bonding and electronic structure of these elements. This does come with the drawback of the computational investment required for such extensive calculations. For this reason, most studies (for example ref.^24^) restrict themselves to crystallographically simple tilt boundaries to reduce the size of the simulation to less than about one hundred atoms. However, the boundaries seen in experimentally measured materials are rarely simple or symmetrical (for example, those found in the textures shown in Fig. 1). Therefore, in the present case, the authors push the size of the simulation to the largest computationally possible size in order to examine non-symmetrical “real” grain boundaries, and these are detailed in Fig. 2. The crystallography of the three boundaries chosen for study were derived from the data in Fig. 1. It can be seen from the pair distribution functions (PDF) for the three grain boundaries that there is a significant difference in the local atomic packing between the three boundaries, resulting in a co-ordination number for boundaries A, B and C of 7.83, 5.10 and 5.07 respectively. All are markedly lower than the co-ordination number of the bulk, 12. Another quantifiable difference between the three boundaries is the median interatomic distance. This parameter drops from 3.2 Å for boundary A to 2.7 Å for boundary B, and 3.0 Å for boundary C. These measurements of the local disorder at the boundary indicate that each atom at the boundary is surrounded by a smaller number of more closely spaced atoms compared to the bulk. These local surroundings are investigated further in the following section with a specific emphasis on how the local packing at the boundary effects the electronic structure of solutes that inhabit the grain boundary. The solutes investigated in most detail are Gd and Zn.

## The effect of boundary topology on electronic structure

While the notion of electronegativity is commonly used to describe the nature of chemical bonding, it may fail to reveal the subtle differences between species where the electronegativities are close to each other^26^. Rather than examining only the charge transfer, in the present work we examine the full density of states. Since the grain boundary is a region of localized disorder, this local disorder will effect the electronic structure of atoms located at the boundary, and this has not before been studied in detail.

**Figure 3:**
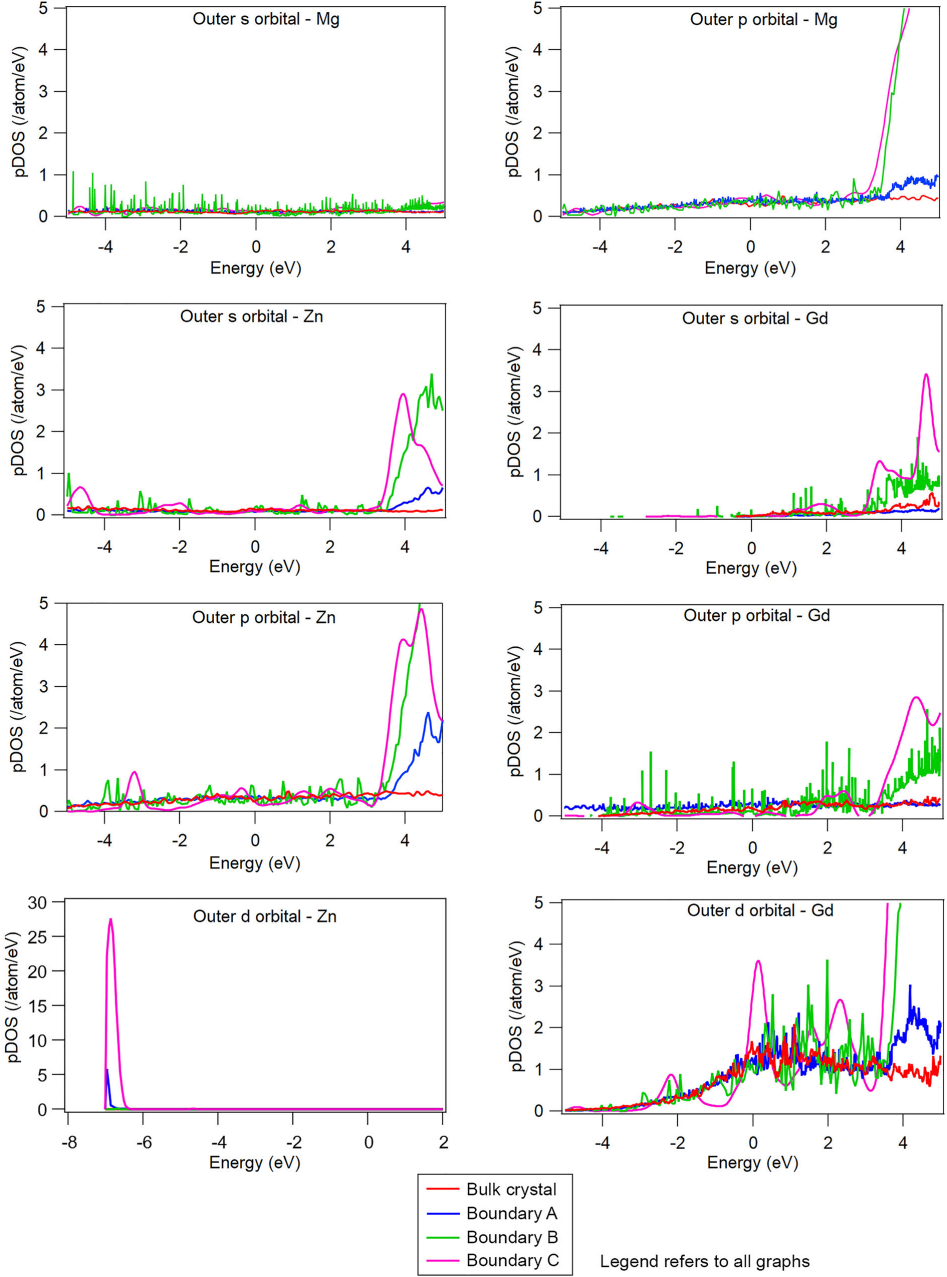
pDOS for Mg, Zn and Gd at four different locations: in the bulk, and at grain boundaries (**A**–**C**). Note the different x-axis scale and y-axis for the d-orbital in Zn.

Here we quantify the changes that occur in the electronic structure of solutes when located at grain boundaries of different crystallography. Figure 3 shows the projected density of states (pDOS) of Gd and Zn in four different locations: in the bulk, and at grain boundaries A, B and C. For reference, the behaviour of pure Mg is also shown for these same four locations. The density of states of a solid (also called total DOS) is projected on the angular momentum orbitals to provide partial density of states, referred to here as the pDOS. It can be seen that the effect of the local topology on Mg is small, with negligible difference in the pDOS for magnesium when the atom is located within the perfectly ordered bulk, or the complex grain boundary. If we next consider zinc, the pDOS in the bulk is basically flat for both s- and p-orbitals. However, when located at the complex grain boundary there is an increase in the pDOS at high energy levels, but these are far above the Fermi level and unlikely to have a large impact on behavior.

As for the Zn, the d-band is also a dominant bonding band, but in this case the Fermi level lies at the top of the d-band. Consequently, most electronic states in the pDOS lie ~ 7 eV below the Fermi level, Fig. 3. The contribution to bonding is therefore markedly reduced as compared to, for example, Gd.

For the Gd s- and p-orbitals, there is negligible difference in pDOS near the Fermi level for the bulk as compared to the three grain boundaries. However, for the d-orbital of Gd, which dominates the total density of states, some fairly large differences can be observed in the pDOS at the Fermi level. Where boundaries A and B have similar pDOS to the bulk, grain boundary C develops a marked rise in pDOS near the Fermi level, indicating that for this solute in this particular boundary, different bonding behaviour can be expected. This is the first concrete evidence that electronic structure of Gd is different at grain boundaries of different crystallography, and this may begin to explain why this element can effect grain boundary properties: of Gd is more sensitive to its surroundings than other elements. Gadolinium will therefore behave differently depending on the local crystallography. Nevertheless, for all boundaries, the outer shell d band stays partially occupied, with the Fermi level located at the bottom of the d-band as indicated by the large number of states located at ~ 0 eV. The solute concentration at the boundary was found to make small changes to the DOS and Fermi level but had a negligible effect on the grain boundary energy and COHP values. A more detailed analysis of boundary concentration is the subject of a forthcoming publication.

It is also pertinent to note that Zn and Mg showed only small changes in pDOS of the bulk as compared to the different grain boundaries, indicating that these species are less likely to exhibit changes in bonding behavior depending on their surroundings.

## The effect of solute species on bonding strength

The pDOS can be further analysed to quantify the differences in electronic structure between the solute species that were visually evident in Fig. 3. The electronic band structure can be partitioned and the Hamiltonian weighted density of states (or crystal orbital Hamiltonian population, denoted here < –COHP>^27–30^ can be computed. This parameter reveals the nature of chemical bonding, with negative and zero values of < –COHP > representing antibonding and non-bonding states respectively. Of particular importance is its value at the Fermi level, and closely below it, where bonding states imply system stability and antibonding states indicate instability. Table 1 summarises the bonding behvaiour of the solutes at each of the boundaries, and it is seen that although the absolute value of < –COHP > is different between the different boundaries, all show a stable bonding state due to their positive values.

**Table 1:** Summary of the < -iCOHP > values for each solute and grain boundary, larger values of < -iCOHP > represent large bond strengths.

	< –COHP > (per bond)				<–iCOHP > (eV per bond)			
	Bonding behaviour				Bonding strength			
	Pure Mg	Mg–Gd	Mg–Zn	Gd–Zn	Pure Mg	Mg–Gd	Mg–Zn	Gd–Zn
Boundary A	0.12	0.29	0.10	0.33	1.05	1.32	1.30	2.18
Boundary B	0.04	0.20	0.07	NA	0.83	1.12	0.99	NA
Boundary C	0.05	0.54	0.04	NA	0.87	0.99	1.11	NA
Bulk	0.12	0.42	0.09	NA	1.16	1.69	1.23	2.50

NA represents parameters which were not measured in this study.

Once a stable bonding condition has been confirmed, we can move forward and examine the strength of this bond. In this case we use the integral of the crystal orbital Hamiltonian population, < –iCOHP > . Since the < –iCOHP > does not include unoccupied states above the Fermi level (which can only be occupied due to an external field or higher temperatures), the < –iCOHP > at the Fermi level can justifiably be taken as a representation of the strength of bonds^25,27,31,32^.

The < –iCOHP > for each solute and boundary are detailed in Table 1. Let’s firstly consider the change in bonding strength at the three different boundary types of crystallography and compare these to the bulk. The bonding strength of for all three species is lower at the boundary compared to the bulk, a result of the local disorder at the grain boundary. Notably, the magnitude of change is largest for Gd, consistent with the notion that this element is more sensitive than others to its local surroundings.

If we now consider the strength of bonding for the three species, we can see that that the strength of the bond between Mg and Gd is usually higher than it is for Mg and Zn. The < –iCOHP > values shown in Table 1 also indicate that the bond between Gd and Zn is larger than either Mg–Gd or Mg–Zn. This may be an explanation for the synergistic effect of these elements when co-located in the microstructure.

## The “rare earth” effect

**Figure 4:**
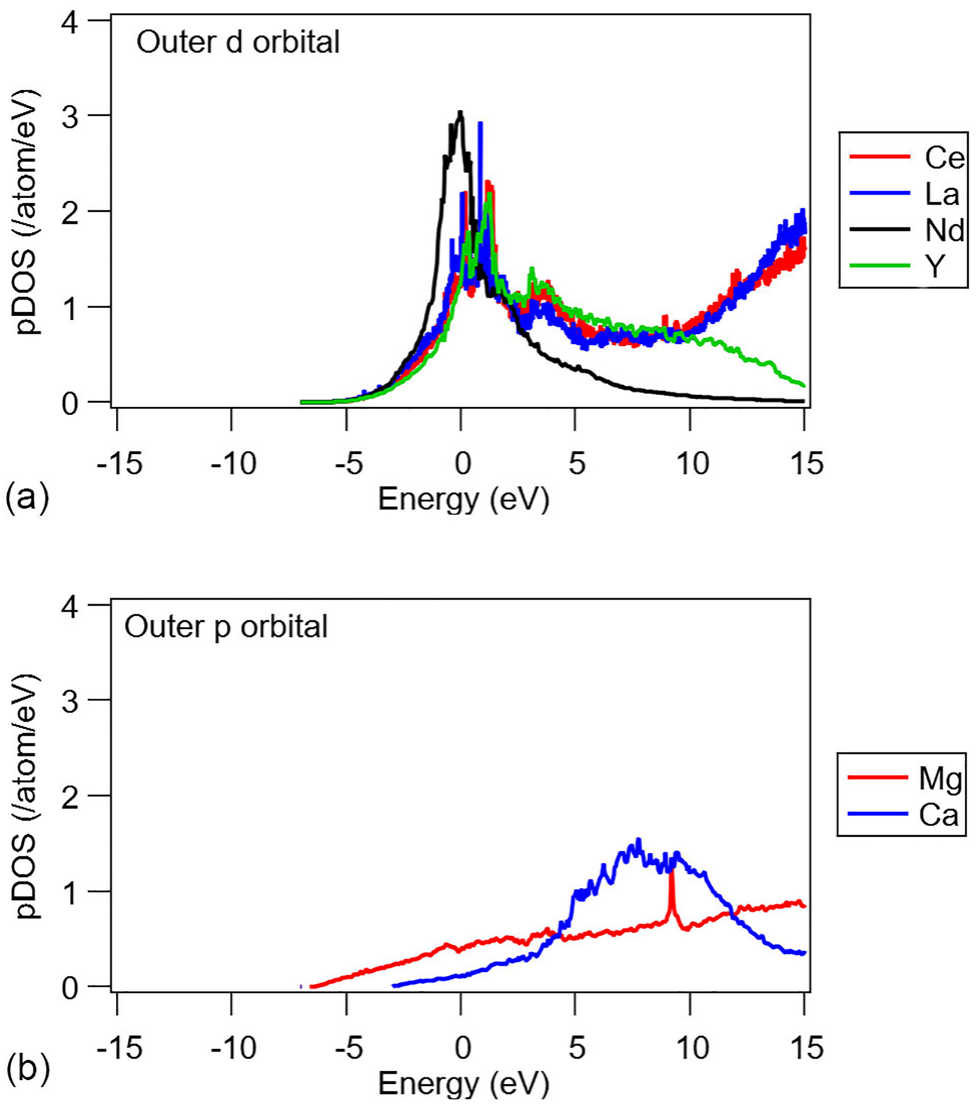
pDOS for selected elements, calculated for solute species located at grain boundary A.

The observation of the differences between Zn and Gd begins to explain the differences in behavior we see in experimental work between different groups in the periodic table. It also somewhat explains why all of the rare-earth elements tend to have similar effects when added to magnesium alloys—they all come from column three of the periodic table and therefore share similar electronic configurations, Fig. 4.

Table 2 summarizes the different bonding behavior and bonding strength values (< –iCOHP >) for a range of elements. It can be seen that the classical texture modifiers, Y and the lanthanides, show stable bonds with a bond strength higher than the Mg–Mg bond. By comparison, Zn and Al, neither of which modify texture in binary alloys, are in quite weakly bonding states with the Mg matrix thereby indicating only marginally stable solid solutions. This is consistent with these elements being outside column three of the periodic table and therefore not containing a valence band energetically structured for strong bonding with Mg. The one outlier in this analysis is Ca. Ca is known to be a texture modifier, albeit, weaker than the lanthanides^33^. Ca is chemically similar to the lanthanides being situated next to them in column 2 of the periodic table. While its s orbital is complete, the p band of Ca is empty and the Fermi level is therefore located at the bottom of the p band (most electronic states lie at or near the Fermi level). Indeed, the RE elements and Ca share the feature that their outer shells have partially occupied bands, and all have their Fermi level located at the bottom of their outer shell band, resulting in a large density of states near the Fermi level at 0 eV. This phenomenon is reflected in their higher values of < –COHP > in the vicinity of the Fermi level indicating more stable solid solution states than Al and Zn.

**Table 2:** Summary of the < –COHP > and < –iCOHP > values at the Fermi level, calculated for pairs of Mg-solutes with interatomic distances of 4 Angstrom or less, within the bulk.

Element	< –COHP > (per bond)	< –iCOHP > (eV/bond)
	Bonding behaviour	Bonding strength
Mg	0.12	1.16
Gd	0.42	1.69
Ce	0.36	2.34
La	0.37	2.40
Y	0.39	2.06
Ca	0.11	1.01
Nd	0.31	2.65
Gd-Zn	0.42	2.50
Al	0.04	1.24
Zn	0.09	1.23

Positive values of < COHP > indicate a bonding state, negative values indicate a non-bonding (anti-bonding) state. Larger values of < –iCOHP > represent larger bond strengths. Note that data for Gd and Zn is repeated from Table 1.

## Closing remarks

It has been argued here that the change in properties that result from RE elements located at the grain boundary is the result of the interplay between the electronic structure of the lanthanide solutes and the local atomic arrangement in the vicinity of a given grain boundary. On the one hand the outer shell of the lanthanide solute is partially occupied, and the Fermi level lies at the bottom of this shell, leading to strong chemical bonding with magnesium. On the other hand, the marked disruptions in the local atomic packing of atoms at the disordered grain boundaries, quantified by local PDF’s, have been shown to significantly modify the electronic structure of solutes located at those boundaries. This effect was found to vary significantly from one boundary to another, and the change in bonding strength (as quantified by < –iCOHP >) was mainly found to be larger for the RE elements, and lower for those elements that do not modify texture (the non-RE elements). These findings show for the first time that the RE elements are bound more strongly to grain boundaries than other solutes, and it is suggested that the preference for these elements to remain strongly bonded at the grain boundary will change other important grain boundary properties such as grain boundary energy and cohesion. The most important property for texture development is grain boundary mobility, and this too is likely to be affected by solutes strongly bonded to the boundary. Although the present study focused only on studying the behaviour of different solutes at static boundaries, mobility predictions based on the current data set are the subject of a future publication which will further interrogate this hypothesis.

It is commonly argued that it is the atomic size of the RE elements that is the underlying feature that gives them unique properties in magnesium. The present quantum mechanical calculations are not inconsistent with this assertion. The large atomic radius of the RE elements make it energetically favorable for RE elements to be located at grain boundaries, while the sensitivity to the boundary crystallography and enhanced bonding can be traced back to the electronic structure, most notably, of the outer electron orbital.

## Materials and methods

The simulations were carried out using the Vienna ab initio software package (VASP)^34^ implementing the projector augmented wave method to represent the combined potential of core electrons and nuclei^35^. The Perdew–Burke–Ernzerhof gradient approximation was implemented to represent the exchange–correlation functional^36^. A cut-off energy of 400 eV was chosen for the plane wave basis and the self-consistent electronic optimization was converged to 10^–6^ eV. The mesh of $\Gamma$-centered k-points to sample the Brillouin zone were chosen such that their density per reciprocal space is at least 50,000 Å^−3^. The atomic configuration is optimized using the conjugate gradient method until the mean atomic forces are less than 0.02 eV/Å^−1^.

The simulation cells were designed to comprise two grains and a large enough vacuum layer on top to prevent interference from the out-of-plane images due to periodic boundary conditions^24^. In order to meet the required periodic boundary condition in the grain boundary plane, without rendering the ab initio simulation unfeasible due to an overly large number of atoms in the cell, the top grain was strained in the boundary plane if required. Details about the simulation cell are given in Table 3.

**Table 3:** Details of the misorientation angle, misorientation axis and cell size of the three grain boundaries.

Grain number		Boundary plane and parallel directions	Axis and angle of misorientation (three-digit indices)	Equivalent four-digit rotation axis	Cell dimension ($\dot{\boldsymbol{A}}$)	Number of atoms in simulation	ϒ_GB_ (J/m^2^)
Boundary A	Grain A1	$\left(0001\right)\langle11\bar{2}0\rangle$	58.31º $\langle\frac{-\sqrt{3}}{2},{0.5,0}\rangle$	$\left[10\bar{1}0\right]$ tilt	5.56 $\times$ 6.42 $\times$ 99.51	128	0.584
	Grain A2	$\left(11\bar{2}2\right)\langle\bar{11}21\rangle$					
Boundary B	Grain B1	$\left(10\bar{1}0\right)\langle0001\rangle$	43.37º $\langle0.52,-{0.52,0.67}\rangle$	irrational	5.21 $\times$ 57.78 $\times$ 52.96	536	0.205
	Grain B2	$\left(11\bar{2}2\right)\langle\bar{11}21\rangle$					
Boundary C	Grain C1	$\left(10\bar{1}0\right)\langle0001\rangle$	61.59º $\langle\frac{\sqrt{3}}{2},{0.5,0}\rangle$	$\left[10\bar{1}0\right]$ twist	46.90 $\times$ 25.68 $\times$ 32.10	977	0.445
	Grain C2	$\left(10\bar{1}0\right)\langle1\bar{2}11\rangle$					

The grain boundary energy (γ_GB_) is also shown.

While the atomic representation of twin boundaries comprises a supercell containing two misoriented grains with a large enough vacuum layer on top^37–40^, the general grain boundary can be considered as a general interface between two grains (G_1_ and G_2_) represented by two slabs. As a consequence, the simulation cell total energy is the contribution of the bulk energy of grains (${E}_{Bulk, G1}$ and ${E}_{Bulk,G2}$), two surface energies (${\sigma }_{G1+}{\sigma }_{G2}$) and the grain boundary energy (${\gamma }_{GB}$) itself. Thus, the latter can be expressed as ${\gamma }_{GB}=$ ${E}_{GB}/S-{(\sigma }_{G1}+{\sigma }_{G2})-({E}_{Bulk, G1}-{E}_{Bulk, G2})/S$ and rearranging the right hand side of this equation will give^41^:

1 $${W}_{sep}=({E}_{FS}^{G1}+{E}_{FS}^{G2}-{E}_{GB})/S$$

2 $${\gamma }_{GB}={\sigma }_{G1}+{\sigma }_{G2}-{W}_{sep}$$

where W_SEP_ and S are the work of separation defined as the reversible work needed to separate the grain boundary into two free surfaces^42^ and boundary area respectively.

When solute is added to the grain boundary, the segregated grain boundary energy can be expressed as ${\gamma }_{GB}^{S}={\gamma }_{GB}+\Delta {E}_{GB}^{S}{\Gamma }$ where $\Delta {E}_{GB}^{S}$ and ${\Gamma }$ are the change in segregation energy of the grain boundary and the conversion factor of segregation energy into energy per area respectively^43^.
